# Structural and Biophysical Analyses of Human N-Myc Downstream-Regulated Gene 3 (NDRG3) Protein

**DOI:** 10.3390/biom10010090

**Published:** 2020-01-06

**Authors:** Kyung Rok Kim, Kyung A. Kim, Joon Sung Park, Jun Young Jang, Yuri Choi, Hyung Ho Lee, Dong Chul Lee, Kyung Chan Park, Young Il Yeom, Hyun-Jung Kim, Byung Woo Han

**Affiliations:** 1 Research Institute of Pharmaceutical Sciences, College of Pharmacy, Seoul National University, Seoul 08826, Korea; krkim85@snu.ac.kr (K.R.K.); lovekka1740@snu.ac.kr (K.A.K.); wingpjs@snu.ac.kr (J.S.P.); nosvc4@snu.ac.kr (J.Y.J.); 2 Department of Chemistry, College of Natural Sciences, Seoul National University, Seoul 08826, Korea; yuri93@snu.ac.kr (Y.C.); hyungholee@snu.ac.kr (H.H.L.); 3 Immunotherapy Research Center, Korea Research Institute of Bioscience and Biotechnology, Daejeon 34141, Korea; dclee@kribb.re.kr (D.C.L.); yeomyi@kribb.re.kr (Y.I.Y.); 4 Personalized Genomic Medicine Research Center, Korea Research Institute of Bioscience and Biotechnology, Daejeon 34141, Korea; kpark@kribb.re.kr; 5 Laboratory of Molecular and Stem Cell Pharmacology, College of Pharmacy, Chung-Ang University, Seoul 06974, Korea

**Keywords:** NDRG3, α/β-hydrolase fold, crystal structure, unfolded helix

## Abstract

The N-Myc downstream-regulated gene (NDRG) family belongs to the α/β-hydrolase fold and is known to exert various physiologic functions in cell proliferation, differentiation, and hypoxia-induced cancer metabolism. In particular, NDRG3 is closely related to proliferation and migration of prostate cancer cells, and recent studies reported its implication in lactate-triggered hypoxia responses or tumorigenesis. However, the underlying mechanism for the functions of NDRG3 remains unclear. Here, we report the crystal structure of human NDRG3 at 2.2 Å resolution, with six molecules in an asymmetric unit. While NDRG3 adopts the α/β-hydrolase fold, complete substitution of the canonical catalytic triad residues to non-reactive residues and steric hindrance around the pseudo-active site seem to disable the α/β-hydrolase activity. While NDRG3 shares a high similarity to NDRG2 in terms of amino acid sequence and structure, NDRG3 exhibited remarkable structural differences in a flexible loop corresponding to helix α6 of NDRG2 that is responsible for tumor suppression. Thus, this flexible loop region seems to play a distinct role in oncogenic progression induced by NDRG3. Collectively, our studies could provide structural and biophysical insights into the molecular characteristics of NDRG3.

## 1. Introduction

The α/β-hydrolase fold superfamily is one of the largest groups of structurally related proteins since its discovery in 1992 [1]. This fold contains eight canonical β-strands surrounded by α-helices, wherein the second strand is antiparallel to the rest. Hydrolytic activity is the main function of the α/β-hydrolase fold superfamily and a nucleophile-histidine-acid in the catalytic triad is essential for their activity. Some α/β-hydrolase fold proteins contain additional motifs with different sizes, structures, and positions, which aid the protein with substrate selection, regulation of hydrolysis, or nonenzymatic function. In particular, nonenzymatic α/β-hydrolase fold proteins, such as neuroligins, gliotactin, and thyroglobulin, have been reported to associate to multiprotein scaffolds or regulate protein–protein interaction [2,3]. In spite of the structural conservation of an active site pocket among the nonenzymatic α/β-hydrolase fold, substitutions of catalytic residues to non-reactive residues in this pocket result in losses of their hydrolytic function. These inactive subfamilies appear to have diverged from a common hydrolase ancestor involving the mutation of the catalytic triad to non-reactive residues [4].

**Figure 1:**
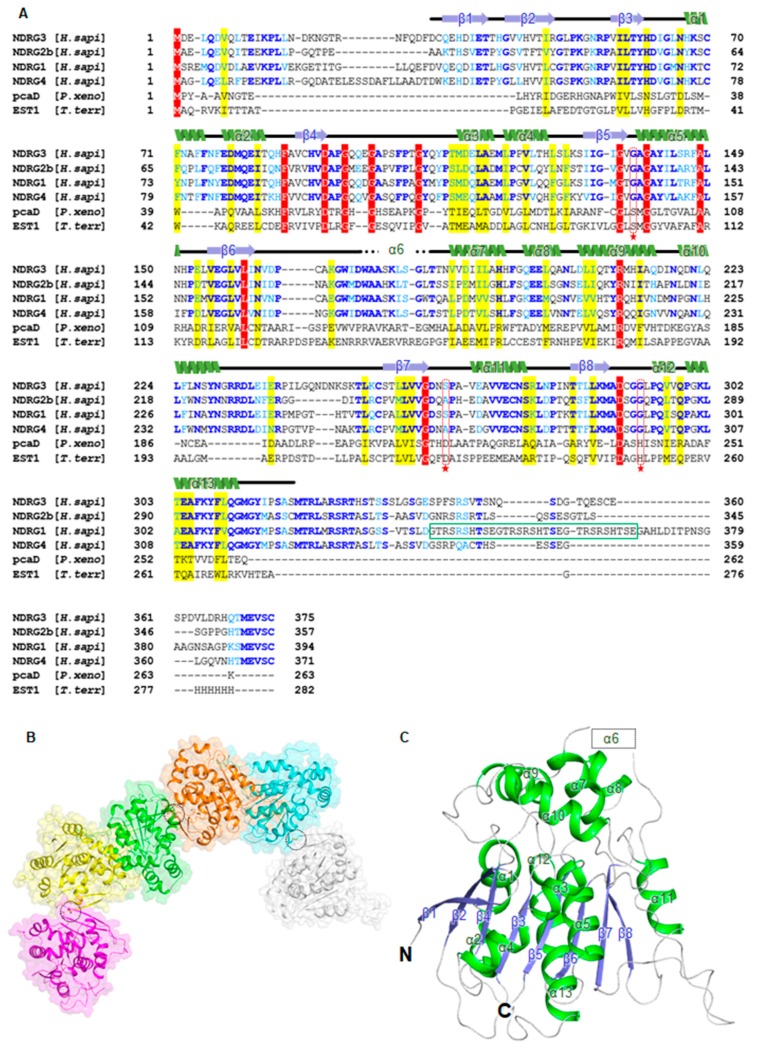
Crystal structure of truncated NDRG3 (residues 29–320, NDRG3 ΔNC). (**A**) Sequence alignment of human NDRG isoforms and structurally similar α/β-hydrolase fold proteins. The secondary structure of NDRG3 is shown above the sequence alignment. The conserved catalytic triads are marked with red asterisks. Strictly and moderately conserved residues are highlighted with red-shaded boxes, and yellow-shaded boxes, respectively. Blue and cyan-colored residues represent the identical and conserved residues among the NDRG isoforms, respectively. The green box indicates the three decapeptide sequence (GTRSRSHTSE) repeats of NDRG1. Sequences were aligned using the T-Coffee web server [30]. (**B**) NDRG3 ΔNC molecules observed in an asymmetric unit of the crystal. Intermolecular disulfide bonds are shown in stick models and marked with black-dotted circles. (**C**) NDRG3 ΔNC structure is shown in cartoon representation. α-helices, β-strands, and loops are colored in green, blue, and white, respectively. The disordered region corresponding to helix α6 of NDRG2 is shown with a black-dashed box and labeled as the helix α6.

The N-Myc downstream-regulated gene (NDRG) family is a member of the α/β-hydrolase superfamily and is repressed by Myc expression [5,6]. Given that Myc overexpression is related to cell proliferation and metastasis, the genes repressed by Myc are believed to regulate tumor progression [7,8]. There are four isoforms of NDRG in humans: NDRG1, NDRG2, NDRG3, and NDRG4. These four proteins share 58–66% sequence identity and NDRG1 contains a characteristic three decapeptide sequence repeats motif in the C-terminal region (Figure 1A and Figure S1A) [9]. While the NDRG family shares a high sequence identity, their tissue distributions differ and they play different roles in tumor regulation: NDRG1 has been proposed as a prognostic biomarker for colorectal cancer because it was reported to suppress cell invasion, migration, and proliferation [10,11,12]. NDRG2 has been known to remarkably reduce cell proliferation in various types of cancer [13,14,15,16,17,18,19], as well as to inhibit signaling pathways of oncogenic factors, such as lymphoid enhancer factor/T-cell factor [20], nuclear factor-κB [21], and matrix metalloproteinase-3 [22]. Any roles of NDRG4 in tumors remain unidentified.

Compared with the tumor suppressive NDRG members, NDRG3 has been reported to be oncogenic. NDRG3 is upregulated in epithelial prostate cancer cells and prostatic stromal cells at both mRNA and protein levels, and overexpression of NDRG3 induces cell proliferation and migration [23]. Interestingly, NDRG3 plays a role in cell proliferation and anti-apoptosis under hypoxia. Lee at al. found a novel ‘NDRG3-Raf-ERK’ pathway mediated by accumulated lactate under hypoxic conditions [24]. Under the normoxia condition, NDRG3 is degraded by prolyl hydroxylases (PHD) 2/Von Hippel–Lindau (VHL) dependent ubiquitination. However, NDRG3 bypasses the degradation pathway when it is complexed with lactate, the end-product of the anaerobic metabolism. The NDRG3-lactate complex is deposited in the cell and directly induces the phosphorylation of c-Raf, triggering ERK-mediated cell proliferation. Furthermore, the lactate-mediated ‘NDRG3-Raf-ERK’ pathway facilitates double-strand break repairs in spermatogenesis by upregulation of RAD51 via its phosphorylating cAMP response element-binding protein (CREB) [25].

In lieu of the lactate-mediated NDRG3 signaling, NDRG3 provokes anti-apoptotic processes during the hypoxia postcondition by regulating adenosine A2a receptors [26], activates Src phosphorylation in colorectal cancer progression [27], and promotes hepatocellular carcinoma metastasis via regulating turnover of β-catenin [28]. On the other hand, NDRG3 is involved in anti-metastatic functions, by dissociating the coactivator p300 from HIF-1a [29]. While NDRG3 has recently received attention for its promiscuous roles in regulating cell proliferation and metastasis, the structure of NDRG3 has not been elucidated yet. To gain insights into the molecular basis of NDRG3, we determined the crystal structure of human NDRG3. Based on our structural analyses of NDRG3, we highlight unique structural features of NDRG3 compared with α/β-hydrolases including NDRG2.

## 2. Materials and Methods

### 2.1. Cloning, Protein Expression, and Purification of NDRG3

The full-length human NDRG3 gene encoded in pGEX4T-2 plasmid was provided by the Korea Research Institute of Bioscience and Biotechnology (Daejeon, Republic of Korea). The truncated NDRG3 gene (residues 29–320, NDRG3 ΔNC) was amplified by PCR using PrimeSTAR^®^ DNA polymerase (Takara, Kusatsu, Japan) and subcloned in the expression vector, pET-28a(+) (Novagen, Kenilworth, NJ, USA) to produce recombinant protein containing an N-terminal hexahistidine tag (MGSSHHHHHHSSGLVPRGSH). The recombinant plasmid was transformed into BLR(DE3), an *Escherichia coli* strain (Novagen, Kenilworth, NJ, USA), and the cells were cultured in Luria–Bertani medium (Alpha Biosciences, Baltimore, MD, USA) supplemented with 30 μg/mL kanamycin. When the transformed cells were in the mid-log growth phase, 0.5 mM of isopropyl β-D-thiogalactopyranoside (IPTG) was added for induction of NDRG3 ΔNC overexpression. The cells were additionally incubated for 16 h at 293 K, then pelleted by centrifugation at 6000× *g* for 10 min at 277 K and disrupted by sonication in a buffer containing 20 mM 2-amino-2-(hydroxymethyl)propane-1,3-diol (Tris) titrated with hydrochloric acid to pH 7.5, 500 mM sodium chloride, and 35 mM imidazole supplemented with 1 mM phenylmethylsulfonyl fluoride (PMSF). The crude lysate was centrifuged at 36,000× *g* for 50 min at 277 K and the resultant supernatant was loaded onto a nickel-charged HiTrap^™^ Chelating HP 5 ml column (GE Healthcare, Chicago, IL, USA). After washing unbound proteins, the column-bound proteins were eluted by the addition of a buffer containing 20 mM Tris-HCl (pH 7.5), 500 mM sodium chloride, and an imidazole gradient increasing from 35 to 1000 mM. During an imidazole gradient elution, NDRG3 ΔNC protein was divided into monomer and dimer fractions (Figure S1B). NDRG3 ΔNC monomer fractions were collected and the protein was desalted with a buffer containing 20 mM Tris-HCl (pH 8.0), 50 mM sodium chloride, 1% glycerol, and 0.5 mM tris(2-carboxyethyl)phosphine hydrochloride (TCEP) using a HiPrep^™^ 26/10 desalting column (GE Healthcare, Chicago, IL, USA) and further purified using a HiTrap^™^ Q HP 5 ml column (GE Healthcare, Chicago, IL, USA) with a linear gradient from 50 to 500 mM of sodium chloride. The eluted proteins were loaded onto a HiLoad^™^ 16/600 Superdex 200 prep grade column (GE Healthcare, Chicago, IL, USA) equilibrated with 10 mM Tris-HCl (pH 7.5), 150 mM sodium chloride, 1% glycerol, and 0.1 mM TCEP. The purified monomer protein was concentrated to 40 mg/mL using an Amicon Ultra-15 centrifugal filter unit (Merck Millipore, Burlington, MA, USA) for further studies. NDRG3 ΔNC dimer fractions were purified using the same protocol as for the NDRG3 ΔNC monomer (Figure S1C).

### 2.2. Mutagenesis and Purification of NDRG3 ΔNC

The NDRG3 ΔNC mutants (C30S, S255A/N281A, I171M/S176H, and C30S/I171M/S176H) were produced by PCR-based site-directed mutagenesis (PrimeSTAR^®^ HS DNA polymerase; Takara, Kusatsu, Japan). NDRG3 S255A/N281A mutant plasmid was transformed into BLR(DE3) *E. coli* strain (Novagen, Kenilworth, NJ, USA). Each cell containing NDRG3 ΔNC wild type (WT) plasmid and S255A/N281A plasmid was cultured and induced by 0.5 mM of IPTG, and additionally incubated for 16 h at 293 K. The cells were pelleted by centrifugation at 6000× *g* for 10 min at 277 K and disrupted by sonication in a buffer containing 20 mM Tris-HCl pH 7.5, 500 mM sodium chloride, and 35 mM imidazole supplemented with 1 mM PMSF. The crude lysates were centrifuged at 36,000× *g* for 50 min at 277 K and the resultant supernatant was loaded onto a nickel-charged HiTrap^™^ Chelating HP 5 ml column (GE Healthcare, Chicago, IL, USA). After washing unbound proteins, the column-bound proteins were eluted by addition of a buffer containing 20 mM Tris-HCl pH 7.5, 500 mM sodium chloride, and 300 mM imidazole. Then, 0.5 mg of eluted NDRG3 ΔNC WT and S255A/N281A proteins were loaded onto Superdex^™^ 200 Increase 10/300 GL (GE Healthcare, Chicago, IL, USA) pre-equilibrated with 10 mM Tris-HCl pH 7.5, 150 mM sodium chloride, 1% glycerol, and 0.5 mM TCEP at flow rate of 0.75 mL/min using the ÄKTA Pure FPLC system (GE Healthcare, Chicago, IL, USA). NDRG3 C30S, I171M/S176H, and C30S/I171M/S176H mutated plasmids were transformed into SoluBL21^™^, *E*. *coli* strain (Gelantis, San Diego, CA, USA). The overexpression and purification steps were the same as for the purification of NDRG3 ΔNC WT. Each purified monomeric mutant was concentrated to 20 mg/mL using an Amicon ultra-15 centrifugal filter unit (Merck Millipore, Burlington, MA, USA) for further studies.

### 2.3. Crystallization

The human NDRG3 ΔNC (residues 29–320) was diluted to 20 mg/mL, and initial crystallization experiments were carried out with commercially available crystal screening kits using the sitting-drop vapor diffusion method at 295 K. First crystals were obtained by mixing 0.5 μL of 20 mg/mL NDRG3 ΔNC and 0.5 μL of a solution containing 200 mM sodium citrate tribasic dihydrate and 20% polyethylene glycol (PEG)3,350 (Index; Hampton Research, Aliso Viejo, CA, USA). Since the crystals did not well diffract, we optimized the crystals with a matrix screening containing 100–300 mM sodium citrate tribasic dihydrate and 10–30% PEG3,350 using the hanging-drop vapor diffusion method. The best diffracting crystal was grown in a drop mixed with 1 μL of 10 mg/mL protein solution, 0.8 μL of a crystallization solution containing 200 mM sodium citrate tribasic dihydrate and 20% PEG3,350, and 0.2 μL of the crystallization solution containing microseeds of the initial crystals. Initial crystals of NDRG3 C30S were obtained with the same crystallization solution as for NDRG3 ΔNC crystals. The best diffracting crystal of C30S mutant was obtained by mixing 15 mg/mL protein supplemented with 0.01 mM TCEP. First crystals of NDRG3 I171M/S176H were obtained by mixing 0.5 μL of 13 mg/mL protein and an equal volume of crystallization solution containing 200 mM ammonium citrate tribasic (pH 7.0) and 20% PEG3,350 buffer condition (PEG/Ion 2; Hampton Research, Aliso Viejo, CA, USA). The crystals of I171M/S176H mutant were optimized with the crystallization solution supplemented with 3% dextran sulfate sodium salt (Mr 5000). All crystals were cryoprotected with paratone oil, then flash-frozen in a liquid nitrogen gas flow at 100 K prior to data collection.

### 2.4. X-ray Data Collection, Refinement and Structure Determination

X-ray diffraction data of NDRG3 ΔNC and C30S mutant crystals were collected at synchrotron beam line BL-7A at the Pohang Light Source (Pohang, Republic of Korea), using a Quantum Q270 CCD detector (Area Detector Systems Corporation, Poway, CA, USA). While the HKL2000 program [31] predicted the structure of the crystals in hexagonal symmetry, we processed and scaled the data using *C*-centered monoclinic symmetry with six monomers in an asymmetric unit (ASU) to describe the disulfide bonds between each Cys30 and dimer. The structure was solved using molecular replacement method with the structure of human NDRG2b protein (PDB Id: 2XMQ) [32] as a searching model using PHASER-MR in the PHENIX software [33]. The model was completed by iterative cycles of refinement using REFMAC5 [34] in the CCP4i software suite [35] and Wincoot [36]. All refinement steps were monitored with R_free_ value calculated from 5.0% of the independent reflections. Because of merohedral twinning of NDRG3 ΔNC crystal, the intensity-based twin law option in REFMAC5 was applied for all refinement processes. The space group of NDRG3 C30S crystal was P3_1_21 and it contains four molecules in an ASU. The crystal structure of NDRG3 C30S was determined using PHASER-MR in the PHENIX software and refined using REFMAC5. We adjusted the TLS refinement option of which parameters were calculated by PDB-REDO [37]. X-ray diffraction data for NDRG3 I171M/S176H were collected at BL-11C Pohang Light Source (Pohang, Republic of Korea), using a Pilatus3 6M detector (Dectris, Baden-Daettwil, Switzerland). The space group of the mutant crystal was P3_2_21 and the structure was determined using PHASER-MR in the PHENIX software. Since the diffraction data were predicted to contain merohedral twinning, twin law was adjusted to mutant refinement at the processing in REFMAC5 as for the NDRG3 ΔNC WT crystal. The stereochemical qualities of the NDRG3 ΔNC and mutants models were checked using MolProbity [38]. The data collection and refinement statistics are summarized in Table 1. Graphical representations for the protein structure were drawn using PyMOL [39]. Coordinates and structure factors are deposited in the Protein Data Bank under accession codes 6L4B (NDRG3 ΔNC), 6L4G (I171M/S176H mutant), and 6L4H (C30S mutant).

### 2.5. Size-Exclusion Chromatography with Multi-Angle Light Scattering (SEC-MALS) Analysis

SEC-MALS was implemented with an FPLC machine (GE Healthcare, Chicago, IL, USA) connected to a Wyatt MiniDAWN TREOS MALS instrument and a Wyatt Optilab rEX differential refractometer (Wyatt Technology, Santa Barbara, CA, USA). A HiLoad^™^ 10/300 Superdex 200 GL (GE Healthcare, Chicago, IL, USA) column was pre-equilibrated with a buffer containing 20 mM Tris-HCl pH 7.5, 150 mM sodium chloride, and 0.5 mM TCEP, and was normalized using ovalbumin. 100 μL of monomer and dimer NDRG3 ΔNC at 2.0 mg/mL were injected into the machine at flow rate of 0.4 mL/min, respectively. Data were analyzed using the Zimm model for fitting static light-scattering data and graphed using EASI graph with a UV peak in the ASTRA V software (Wyatt Technology, Santa Barbara, CA, USA).

### 2.6. Circular Dichroism (CD)

CD spectroscopy was implemented with the Chiranscan^™^-plus CD Spectrometer (Applied photophysics Ltd., Surrey, UK) at 298 K with a wavelength range from 260 nm to 180 nm. NDRG3 ΔNC, NDRG3 mutants, and NDRG2 proteins were diluted to 0.4 mg/mL with a buffer containing 20 mM potassium phosphate dibasic pH 7.5 and 50 mM sodium fluoride. The maximum absorbances in CD wavelength were adjusted to have ranges from 0.80 to 0.85. The bandwidth was 1.5 nm and the time per point value was 0.5. The temperature was set to 298 K.

## 3. Results

### 3.1. Overall Structure of Human NDRG3 Contains an α/β-Hydrolase Fold Domain and a Cap-Like Domain

The human NDRG3 protein (375 amino acids) was predicted to contain flexible N- and C-terminal regions by the Xtalpred server (http://ffas.burnham.org/XtalPred-cgi/xtal.pl) [40]. Additionally, the known post-translational modification (PTM) data from the PhosphoSitePlus database (https://www.phosphosite.org) [41] indicated that the C-terminus of NDRG3 contains numerous phosphorylation sites, suggesting that the C-terminal region is highly dynamic. We tried to crystallize not only full-length NDRG3 but diverse truncated constructs. Among them, a truncated construct (residues 29–320, NDRG3 ΔNC) with an N-terminal hexahistidine tag was successfully crystallized. The NDRG3 ΔNC structure was determined at 2.2 Å resolution using the molecular replacement method with the crystal structure of human NDRG2b (PDB ID: 2XMQ) as a search model, that shares 59.6% sequence identity [32]. The NDRG3 ΔNC crystal contains six monomers in an asymmetric unit (ASU), and belongs to space group C2 (Figure 1B). The NDRG3 ΔNC structure includes two domains: A canonical α/β-hydrolase fold domain and a cap-like domain. The α/β-hydrolase fold domain consists of an eight-stranded β-sheet and eight α-helices (α1–α5 and α11–α13). The β-hairpin structure (β1 and β2) is exposed to the surface, while six parallel β-strands (from β3 to β8) are surrounded by α-helices (Figure 1C and Figure S1D). The cap-like domain (from Ala167 to Arg233) contains a disordered region in six subunits and four helices (α7–α10), which compactly cover the α/β-hydrolase fold by interacting with four loops. The disordered region in the cap-like domain (from Trp173 to Leu182) is sequentially matched to the helix α6 region in NDRG2b (Figure 1A). Although we could not model the region (from Trp173 to Leu182), we will designate the disordered region as the helix α6 region in accordance with the secondary structure of NDRG2b for the sake of convenience. When we compared root-mean-square deviations (r.m.s.d.) distances of C_α_ atoms of each subunit compared with chain A as a reference, overall subunits were structurally similar and the helix α6 region is disordered in all the subunits. Interestingly, helix α8 of chain D, E, and F were structurally different from that of chain A. In the crystal packing of NDRG3 ΔNC, helix α8 of chain D, E, and F were influenced by adjacent molecules while that of chain A, B, and C were away from adjacent molecules. Therefore, the crystal structure of chain A, B, and C of NDRG3 ΔNC seems to represent the structure of NDRG3 in solution than that of chain D, E, and F (Figure S1E,F).

### 3.2. Crystal Packing of NDRG3 Structure Indicates Dimeric Interactions

**Figure 2:**
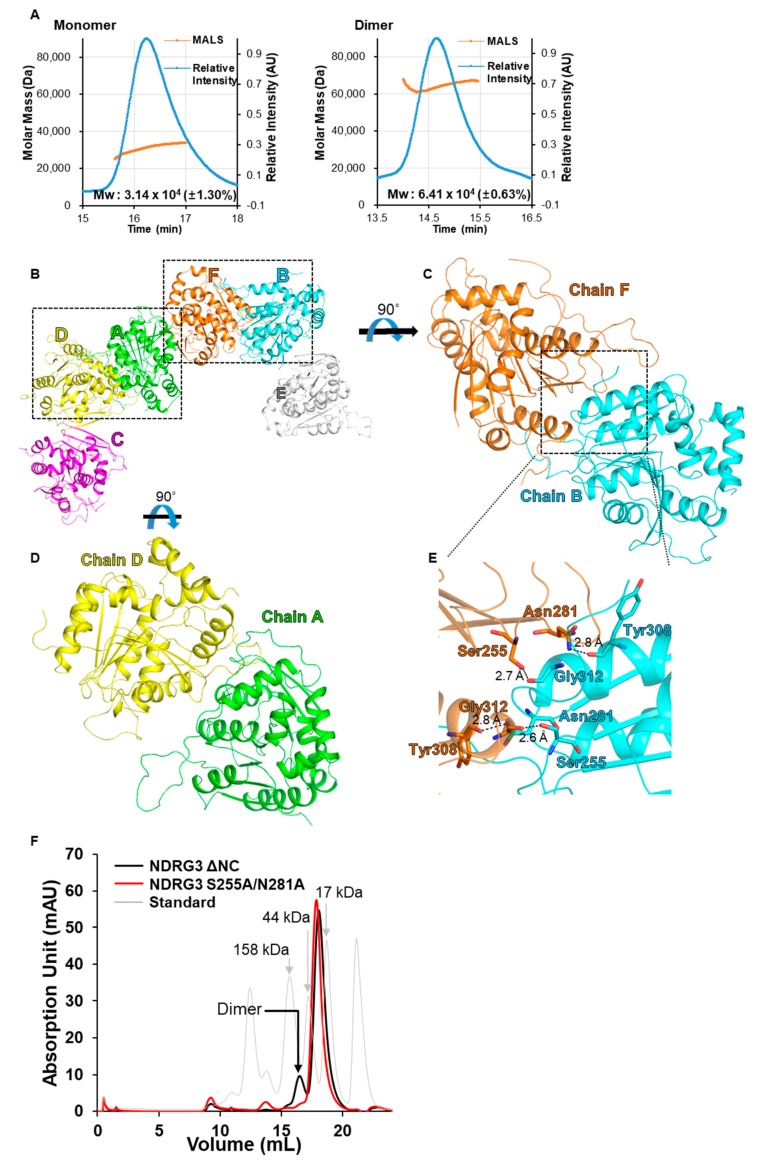
Dimeric interactions of NDRG3 ΔNC. (**A**) SEC-MALS analyses of the NDRG3 ΔNC monomer and dimer. The blue line indicates the relative absorbance of protein during size-exclusion chromatography, and the orange line represents the mass of the molecules analyzed by MALS. (**B**) A hexamer structure of NDRG3 ΔNC in an asymmetric unit (ASU). Each NDRG3 ΔNC structure is represented as a cartoon model in different colors and chain numbers are denoted as letters with the same color. (**C**) Dimeric interactions of chain B and chain F in an ASU. (**D**) Dimeric interactions of chain A and chain D in an ASU. (**E**) A close-up view of hydrogen bonds between chain B and chain F. Residues involved in hydrogen bonding were shown in stick models with labels. (**F**) Chromatograms of NDRG3 ΔNC and S255A/N281A from analytical size exclusion chromatography. The grey chromatogram indicates a gel filtration standard profile (Bio-Rad #1511901, Hercules, CA, USA).

During purification, NDRG3 ΔNC exists as both monomer and dimer in solution, though it was mainly eluted as monomer fraction. To date, the NDRG3 dimer has not been reported. To further investigate the oligomeric state of NDRG3 in solution, we implemented size-exclusion chromatography with multi-angle light scattering (SEC-MALS). SEC-MALS results indicated that the molecular weight of monomer and dimer fractions of NDRG3 ΔNC was calculated as 31.4 kDa and 64.1 kDa, respectively (Figure 2A). While the molecular weights are approximately 10% less than predictions, the dimer is stable in solution. In the crystal structure of NDRG3 ΔNC, the crystal contains six molecules connected by disulfide bonds between Cys30–Cys30 in an ASU, whereas it was grown with monomer fractions in purification (Figure 2B). Since the SEC-MALS was implemented under a reducing agent, 0.5 mM TCEP, the disulfide bond between Cys30–Cys30 does not seem to contribute forming dimer in solution, but it is a critical interaction for forming crystals. To our surprise, the crystal structure contains two different dimeric interactions: Chain A/D and chain B/F (Figure 2C,D) in an ASU. The crystallographic interface of chain C and chain E is very similar to that between chain A and chain D. The Protein, Interfaces, Structures, and Assemblies (PISA) web server [42] predicted that the interface between chain B and chain F comprises a similar area (971.5 Å^2^) as that of the interface between chain A and chain D (999.3 Å^2^). However, the predicted solvation free energy of the dimeric interface between chain B and chain F was −11.5 kcal/mol, which is more stable than that of chain A and chain D (−4.8 kcal/mol). To clarify the dimeric interface of NDRG3 ΔNC, we mutated Ser255 and Asn281 which are key residues involved in hydrogen bond interactions between chain B/F (Figure 2E). After each cells containing NDRG3 ΔNC wild type (WT) plasmid and S255A/N281A mutant plasmid were overexpressed in a same condition, we compared the ratio of dimer/monomer fractions between NDRG3 ΔNC WT and S255A/N281A. The dimer fraction of NDRG3 S255A/N281A was noticeably decreased to 2% out of the total proteins, while the dimer fraction of NDRG3 ΔNC WT was 12% (Figure 2F). Taken all together, our results indicate that NDRG3 ΔNC forms a dimer, wherein the chain B/F dimer represents dimeric interactions of NDRG3 ΔNC.

### 3.3. NDRG3 Shows a Structural Similarity to NDRG2 and Contains a Distinctive Disordered Region and a Solvent Accessible Cavity

To gain an insight into structural features of NDRG3, we analyzed the structural similarities using the DALI web server [43]. The results showed that the structure of NDRG3 ΔNC is similar to α/β-hydrolase fold superfamily. With an exception of NDRG2b structures, NDRG3 ΔNC shares a high similarity with α/β-hydrolases marked with Z score 9.5–26.9, whereas the amino acid sequence alignment shows a low similarity (7–20%). When comparing the structure of NDRG3 ΔNC to the closest structural homologs, pcaD enol-lactonase from *Paraburkholderia xenovorans* (PDB ID: 2XUA, Z score: 26.7, sequence identity: 15.6%) and malate complexed esterase (EST) from *Thermogutta terrifontis* (PDB ID: 4UHE, Z score = 26.2, sequence identity = 18.5%), the overall structure of NDRG3 ΔNC was superposed to the α/β-hydrolases with r.m.s.d. of equipositional C_α_ atoms at 2.8 Å and 2.9 Å, respectively (Figure S2A). However, as shown in structural comparisons between NDRG2b and α/β-hydrolase proteins [32], the residues of canonical catalytic triad sites of α/β-hydrolase family: Nucleophile-acid-histidine, are substituted by non-catalytic residues in NDRG3 ΔNC (Figure S2B), and helices α7 and α10 effectively block the binding path of substrates (Figure S2C). Taken together, structural comparisons between NDRG3 ΔNC and α/β-hydrolase proteins demonstrate that NDRG3 abolishes its hydrolase function, and supports that NDRG3 is a nonenzymatic member of the α/β-hydrolase family.

**Figure 3:**
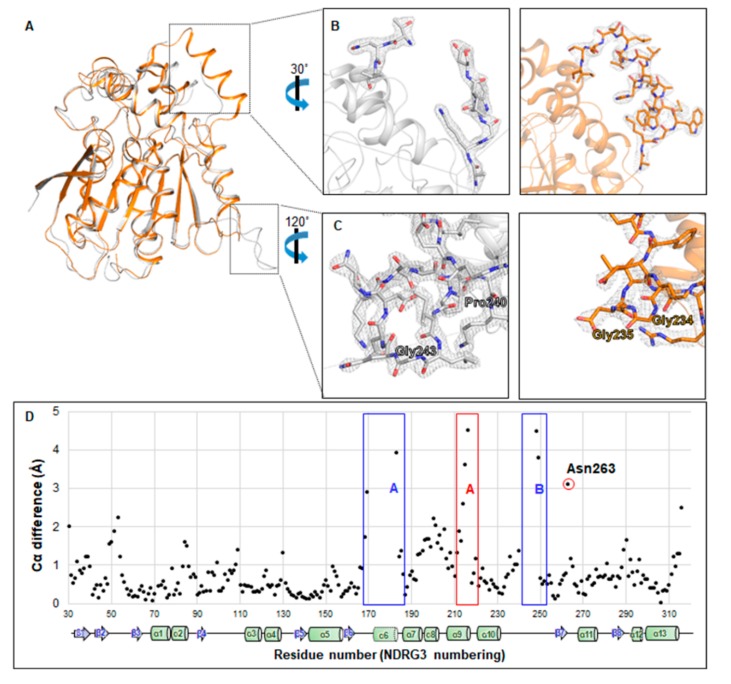
Structural comparison between NDRG3 ΔNC and NDRG2. (**A**) Overall view of superimposition of NDRG3 ΔNC and NDRG2. NDRG3 and NDRG2 structures are represented as white and orange cartoon models, respectively. (**B**,**C**) Electron density map of helix α6 region and loop region between helix α10 and β7 in NDRG3 ΔNC and NDRG2. NDRG3 is represented as a white stick model and NDRG2 is represented as an orange stick model. The 2mFo-DFc electron density map contoured at 1.5σ is represented as a grey-colored mesh. (**D**) Structural comparison of C_α_ distances between NDRG3 ΔNC and NDRG2. The secondary structure of NDRG3 is shown at the bottom of the r.m.s.d. comparison of C_α_. The blue box A denotes the helix α6 region; the blue box B denotes the loop region between helix α10 and β7. The red box A indicates helix α9; the red circle denotes Asn263 on NDRG3.

The first structure determined among the NDRG family proteins, NDRG2, shares a high amino acidic and structural similarity to NDRG3 ΔNC (Figure 1A and Figure 3A). However, there are two regions that show distinctive differences between NDRG2 and NDRG3 ΔNC: The helix α6 region of NDRG2 (Figure 3B) and the loop region between helix α10 and β7 (Figure 3C). While the equivalent region of α/β-hydrolase proteins, pcaD, and EST, adopts a helical structure between helix α10 and β7, it is found as a loop in the structure of NDRG2 and NDRG3 ΔNC. The loop of NDRG2 is composed of charged residues, such that it is exposed to surface, wherein two glycines (Gly234 and Gly235) seem to be critical for formation of the loop. The loop of NDRG3 ΔNC between Gly231 and Ser255 is longer than that of NDRG2 by seven residues, and highly dynamic in that the r.m.s.d. values of C_α_ atoms in the loop region are noticeably higher than the average r.m.s.d. values for all C_α_ atoms, compared with chain A as a reference (Figure S1F). A structural difference of the helix α6 region will be discussed in Section 3.4.

To further investigate the structural difference between NDRG2 and NDRG3 ΔNC, we compared the r.m.s.d. of C_α_ atoms between NDRG2 and NDRG3 ΔNC (Figure 3D). Although they share a high structural similarity with 0.92 Å deviation, helix α9 on NDRG3 ΔNC exhibits a large conformational change by tilting at a 16.0˚ angle difference compared with NDRG2 (Figure S3A). The C-terminal sequence of helix α9 (red box A in Figure 3D), “MHIAQ”, of NDRG3 is aligned to “NIITH” of NDRG2, and helix α9 is an amphipathic helix. In helix α9 of NDRG3 ΔNC, His212 interacted with Asp216 through a hydrogen bond, and the hydrogen bond dragged helix α9 to helix α1. On the other hand, His212 is substituted by Ile206 in NDRG2 which resulted in its being unable to form a hydrogen bond with Ala210. As such, helix α9 in NDRG2 appeared closer to helix α7 than in NDRG3 ΔNC, due to this hydrophobic effect. Moreover, the location of Asn263 is a remarkable point in comparison in terms of the r.m.s.d. of C_α_ from NDRG2 (Figure 3D). Asn263 of NDRG3 ΔNC was found near to loop between β8 and helix α12, forming a hydrogen bond with the main chain of Cys290. On the other hand, Gln250, the corresponding residue in NDRG2, faces to helix α11 and interacts with Glu254 via hydrogen bonding (Figure S3B). Because the Glu254 is substituted to Val267 in NDRG3, Asn263 is unable to interact with helix α10, resulting in the loop between helix α10 and strand β7 bending towards helix α11.

### 3.4. Unfolded Helix α6 Region of NDRG3 Is a Flexible Loop

**Figure 4:**
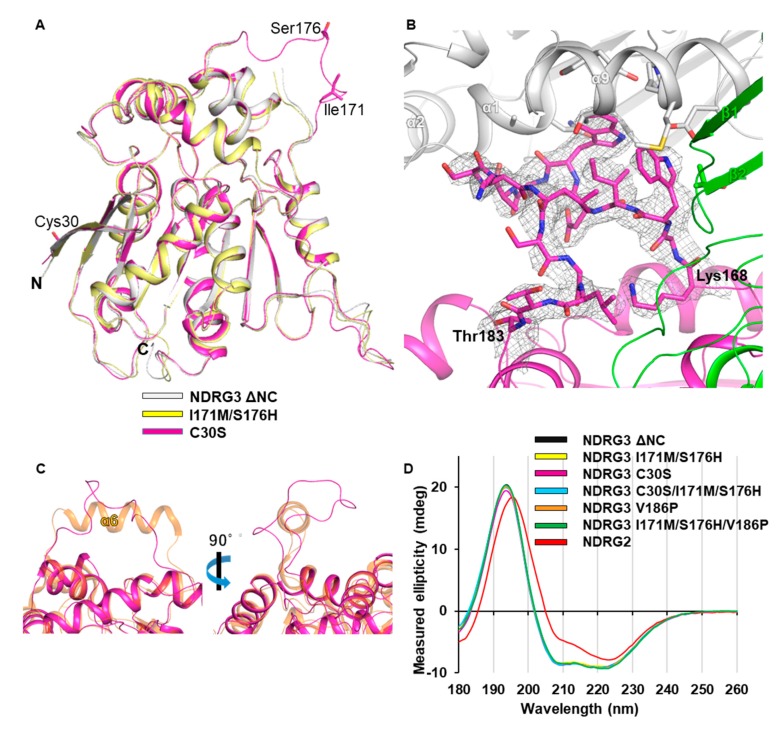
(**A**) Superimposition of the crystal structures of the NDRG3 ΔNC, NDRG3 I171M/S176H, and NDRG3 C30S. Structures of NDRG3 ΔNC, NDRG3 I171M/S176H, and NDRG3 C30S are represented as white, yellow, and magenta cartoon models, respectively. (**B**) Electron density map of the helix α6 region of NDRG3 C30S mutant. Chain A is represented as a magenta ribbon and residues on the helix α6 region are shown as stick models with an electron density map. Chain B and chain C of NDRG3 C30S are shown in green and white cartoon models, respectively. The 2mFo-DFc electron density map contoured at 1.5 σ is represented as a grey-colored mesh. (**C**) Structural comparison of the helix α6 region between NDRG3 C30S and NDRG2. Structures of NDRG3 C30S and NDRG2 are shown in magenta and orange cartoon models, respectively. (**D**) CD spectra of NDRG3 ΔNC, I171M/S176H, C30S, C30S/I171M/S176H, V186P, I171M/S176H/V186P, and NDRG2.

Helix α6 in NDRG2 is known to play a key role in regulating TCF/β-catenin signaling. Interestingly, although NDRG2 and NDRG3 share high similarity in terms of their sequence and structure, the helix α6 region in NDRG3 ΔNC was disordered, whereas that of NDRG2 was presented as a clear electron density map (Figure 3B). In the crystal structure, we suspected that the space between the adjacent molecules is confined and that the helix α6 fold could be disrupted due to the lack of space caused by strong disulfide bonds between each Cys30. To Increase the space for the helix α6 region, we designed a disulfide-deficient, denoted as NDRG3 C30S. The crystal structure of NDRG3 C30S mutant was determined with four monomers in an ASU at 3.4 Å resolution (Figure 4A). Unexpectedly, when the helix α6 region of chain A was modeled alongside its electron density map, the region was a loop and interacted with chain B (Figure 4B). While the intermolecular interaction of the helix α6 region in the C30S mutant was the result of a crystallographic artefact, the region does not seem to form a helix, based on the crystal structure of C30S mutant (Figure 4C). As such, the helix α6 region in NDRG3 ΔNC is a flexible loop in contrast with that of NDRG2 and α/β-hydrolase proteins.

To determine the effect of the residues in forming helix, we constructed a helical wheel model using the helix α6 sequence. The helix α6 model of NDRG3 is amphipathic where the electrostatic surface appears to be analogous to that of NDRG2 (Figure S3C). Comparing helical propensities of each residue on helix α6 between NDRG3 and NDRG2 [44], Ile171 and Ser176 of NDRG3 overlap with Met165 and His170 of NDRG2, respectively. Based on the helical wheel model, we designed NDRG3 I171M/S176H and C30S/I171M/S176H mutants, which mimicked the helix α6 sequences of NDRG2, and determined the crystal structure of the I171M/S176H mutant at 3.3 Å resolution (Figure 4A). The structure of I171M/S176H mutant did not exhibit a clear electron density map in helix α6 region, likewise to that of NDRG3 ΔNC WT.

In the case of the NDRG3 C30S/I171M/S176H, we could not obtain crystals for structure determination, so we implemented circular dichroism (CD) experiments to compare secondary structure elements of NDRG3 ΔNC WT, NDRG3 mutants, and NDRG2 (Figure 4D). While the ellipticities of CD spectrum from NDRG3 ΔNC at 222 nm and 208 nm (θ222 and θ208) were lower than those from NDRG2, the θ222/θ208 ratios of NDRG3 ΔNC and NDRG2 were 1.10 and 1.93, respectively. Considering that θ222/θ208 ratios represent extents of inter-helix interactions [45,46,47], α-helices of NDRG2 are more associated together than those of NDRG3 ΔNC. The CD spectrum of NDRG3 C30S/I171M/S176H was very similar to that of NDRG3 ΔNC WT but not to that of NDRG2. Therefore, NDRG3 C30S/I171M/S176H seems to be structurally similar to NDRG3 ΔNC WT. When we further analyzed sequences before and after the helix α6 region of NDRG3 compared with those of NDRG2, the amino acid sequences following the helix α6 region are TNVV and SSIP in NDRG3 and NDRG2, respectively (Figure 1A). Furthermore, the structures of following the helix α6 region do not superimpose well (Figure 3C). Among residues following the helix α6 region, Val186 in NDRG3 and Pro180 in NDRG2 exhibited the most different biophysical properties. To see whether residues directly following the helix α6 region affect local folding, we mutated Val186 in NDRG3 to Pro and analyzed secondary structure elements using CD. However, CD spectra of NDRG3 V186P and I171M/S176H/V186P mutants did not exhibit changes compared with that of the NDRG3 ΔNC WT (Figure 4D). Taken all together, the unfolded helix α6 region of NDRG3 is structurally unique in comparison to NDRG2 or any other α/β-hydrolase fold proteins, and the structure is not only determined by nearby sequences but seems to be influenced by its surrounding environment.

## 4. Discussion

Since the first characterization of NDRG1 in 1999, the NDRG family has been reported to play promiscuous roles in tumorigenesis or tumor suppression. Then a pro-tumorigenic activity was reported for a member of the NDRG family, NDRG3, when it was known to be associated with prostate cancer [23]. Later, in the last five years of studies have elucidated additional roles of NDRG3 in various hypoxia responses, including cell proliferation, double-strand break repair, metastasis, and HIF regulation [24,25,26,27,28,29]. In this study, we determined the crystal structure of a truncated human NDRG3 fragment comprising residues from 29 to 320 (NDRG3 ΔNC), on the basis of its solubility and crystallizability.

While NDRG3 ΔNC mainly exists as a monomer, we observed a stabilized dimer fraction during purification. SEC-MALS under reducing conditions indicates that the disulfide bond does not seem to contribute to its dimerization in solution. To characterize the dimeric association of NDRG3, it was worth noting that the dimeric interface between chain B/F and chain A/D faced different directions in our crystal structure. As shown in Figure 2C, the dimeric interface of chain B/F is constituted with vis-à-vis each canonical α/β-hydrolase fold domain by covering helix α13 using the α10–β7 loop of NDRG3 ΔNC, comprising 7.5% of total surface area. The dimeric interface area between chain A/D is slightly larger than that of chain B/F (7.7% of total surface area) by interacting between each α10–β7 loop and each helix α10, respectively. However, PISA predicted that the chain B/F dimer is stable in solution, whereas the chain A/D dimer was predicted to result from crystal packing only. While the number of hydrogen bonds was equal between both dimers, the length of the hydrogen bonds in the chain B/F dimer was shorter than that of the chain A/D dimer. In addition, the predicted hydrophobic effect of the dimeric interface between chain B and chain F was −11.5 kcal/mol, which comprises 2.2% of total solvation energy, whereas that between chain A and chain D was −4.8 kcal/mol. Our NDRG3 ΔNC S255A/N281A mutant study further validated the dimeric interface of NDRG3 ΔNC in vitro (Figure 2E,F). When we consider that Ser255 and Asn281 of NDRG3 are unique sequences among NDRG family and NDRG2 exists as monomer [32], dimerization of NDRG3 would be a distinctive feature in comparison with other NDRG family proteins. Further studies should be conducted to elucidate physiological relevance of the NDRG3 dimer.

NDRG3 has been known to be degraded by PHD2/VHL mediated ubiquitination under normoxia condition, and Pro294 hydroxylation of NDRG3 by PHD2 is a critical step of NDRG3 ubiquitination. To get a clue of ubiquitination of NDRG3, we analyzed ubiquitination sites on NDRG3 using data from the PhosphoSitePlus web server (https://www.phosphosite.org) [41]. Our truncated NDRG3 structure contains two ubiquitination sites, K51 and K286, and K286 site was characterized by mass spectrometry in human cell [48], while K51 is a predicted ubiquitination site in human NDRG3. K286 is located in β8 and the region was not well structurally superposed (Figures S1F and S3D). In the case of HIF-1α, a representative protein ubiquitinated by PHD2, Pro402, and Pro564 of HIF-1α is hydroxylated by PHD isoforms, and ubiquitination sites of HIF-1α are close to the Pro residues [49]. Surprisingly, Pro294 of NDRG3 is hydroxylated by PHD2 [24]. Furthermore, K300 and K306 of NDRG1 are known as ubiquitination sites and the Lys residues are amino acidic conserved in NDRG3. Taken together, we suggested that equipositional K301 and K307 of NDRG3 would be novel ubiquitination sites.

Sequence comparisons and structural studies revealed that proteins of the NDRG family contain an α/β-hydrolase fold domain. Although NDRG3 has a high structural similarity to the α/β-hydrolase family, the substitution of the residues of the enzymatic catalytic triad and narrow folded cavity due to the presence of helix α7 and helix α10 indicate that NDRG3 is a nonenzymatic α/β-hydrolase fold protein. A previous report on the crystal structure of NDRG2 revealed the structural features of helix α6 compared with α/β-hydrolase family proteins and showed that helix α6 plays a key role in regulating TCF/β-catenin signaling as a nonenzymatic α/β-hydrolase fold protein. [32]. To our surprise, while NDRG2 and α/β-hydrolase family proteins possess helix α6, the helix α6 region in the crystal structure of NDRG3 ΔNC was disordered, even though the sequences of the helix α6 region in NDRG2 and NDRG3 are similar. Moreover, the crystal structure of NDRG3 C30S, which counteracts the effects of disulfide bonding, and the CD results of NDRG3 ΔNC and NDRG3 C30S mutant provided evidence that the helix α6 region in NDRG3 ΔNC is a flexible loop. Since helix α6 in NDRG2 plays a pivotal role in tumor suppressor activity [32], the unfolded helix α6 region in NDRG3 seems to exert a different function from helix α6 of NDRG2. Further studies would be needed to demonstrate molecular functions of the unfolded helix α6 region of NDRG3.

## 5. Conclusions

We reported the crystal structure of human NDRG3 and demonstrated the dimeric conformation of NDRG3 from crystal packing in an ASU. Compared with NDRG2, the helix α6 region of NDRG3 is a flexible loop. Considering that helix α6 of NDRG2 regulates TCF/β-catenin signaling, the unfolded helix α6 of NDRG3 would play a distinctive role upon interacting with physiologically relevant binding partners. Our structural studies on NDRG3 will shed light on the characterization of NDRG family proteins, providing a fundamental source for understanding the molecular mechanism of NDRG3 in hypoxia responses.

## Figures and Tables

**Table 1:** Statistics for data collection and model refinement.

	NDRG3 ΔNC	NDRG3 C30S	NDRG3 I171M/S176H
**Data Collection ^a^**			
Beamline	PLS-7A	PLS-7A	PLS-11C
Space group	*C*2	*P*3_1_21	*P*3_2_21
Cell dimensions			
a, b, c (Å), α, β, γ (°)	173.34, 100.15, 110.74, 90.00, 90.01, 90.00	99.76, 99.76, 332.71, 90.00, 90.00, 120.00	100.39, 100.39, 111.76, 90.00, 90.00, 120.00
X-ray wavelength (Å)	0.9793	0.9793	0.9794
Resolution (Å) ^b^	50.0–2.2 (2.24–2.20)	50.0–3.4 (3.46–3.40)	50.0–3.3 (3.36–3.30)
<*I*/σ(*I*)>	15.9 (2.5)	16.6 (2.6)	14.0 (3.0)
Unique reflections Redundancy	95,796 (4,774) 5.2 (5.1)	26,798 (1,311) 6.6 (6.7)	10,073 (485) 10.5 (8.8)
Completeness (%)	99.7 (99.3)	97.7 (97.5)	99.3 (95.8)
*R_merge_* (%) ^c^ *R_p.i.m_* (%) ^d^	10.1 (64.1) 4.9 (31.1)	10.3 (73.2) 3.9 (27.5)	16.4 (62.9) 5.4 (21.5)
**Refinement**			
No. of reflections	88,791	25,238	9142
Resolution (Å)	50.0–2.2 (2.24–2.20)	50.0–3.4 (3.46–3.40)	50.0–3.3 (3.36–3.30)
*R*^e^*_work_*/*R_free_*^f^ (%)	16.8%/18.5%	24.1%/27.7%	19.6%/22.6%
Twin fraction	0.172, 0.177, 0.195, 0.129, 0.131, 0.197 ^g^		0.502, 0.498 ^h^
No. of subunits	6	4	2
No. of protein atoms	13,139	8673	4345
No. of solvent atoms	334	0	6
Mean B value (Å^2^)	31.59	125.18	33.22
Ramachandran plot (%)			
favored	1629 (97.7%)	1085 (98.4%)	541 (98.0%)
allowed	39 (2.3%)	18 (1.6%)	11 (2.0%)
outliers	0 (0%)	0 (0%)	0 (0%)
Rotamer outliers (%)	0 (0%)	0 (0%)	0 (0%)
r.m.s. deviations			
bond lengths (Å)	0.002	0.003	0.004
bond angles (°)	1.143	1.246	1.279

^a^ Data collected at the Pohang Light Source; ^b^ numbers in parentheses indicate the highest resolution shell of 20; ^c^ R_merge_ = Σ_h_ Σ_i_ |I(h)_i_ − <I(h)>|/Σ_h_ Σ_i_ I(h)_i_, where I(h) is the observed intensity of reflection h, and < I(h) > is the average intensity obtained from multiple measurements; ^d^ R_p.i.m_ = Σ_h_ √ (1/n − 1) Σ_i_ |I(h)_i_ − <I(h)>|/Σ_h_ Σ_i_ I(h)_i_, where I(h) is the observed intensity of reflection h, and < I(h) > is the average intensity obtained from multiple measurements; ^e^ R = Σ | |F_o_| − |F_c_| |/Σ |F_o_|, where |F_o_| is the observed structure factor amplitude and |F_c_| is the calculated structure factor amplitude; ^f^ R_free_ = R-factor based on 4.9% of the data excluded from refinement; ^g^ Twin operation is (h, k, l), (−h, −k, l), (−1/2*h − 3/2*k, −1/2*h + 1/2*k, −l), (−1/2*h + 3/2*k, 1/2*h + 1/2*k, −l), (1/2*h + 3/2*k, 1/2*h − 1/2*k, −l), and (1/2*h − 3/2*k, −1/2*h − 1/2*k, −l), in order. Twin fractions were calculated by REFMAC5 [34] in the CCP4i software suite [35]; ^h^ the twin operation is (h, k, l) and (−k, −h, −l), in order. Twin fractions were calculated by REFMAC5 [34] in the CCP4i software suite [35].

## Supplementary Materials

The following are available online at https://www.mdpi.com/2218-273X/10/1/90/s1, Figure S1: Sequence identities among NDRG isoforms and topology diagram of NDRG3, Figure S2: Structural comparison between NDRG3 and its structural homologs, pcaD and EST, Figure S3: Structural comparison between NDRG3 and NDRG2.
