# Supplementary material for: Structural and Biophysical Analyses of Human N-Myc Downstream-Regulated Gene 3 (NDRG3) Protein
Source: Biomolecules. 2020 Jan 6;10(1):90. doi: 10.3390/biom10010090 (PMC7022630; doi:10.3390/biom10010090)
Supplement: Supplementary file 1 [file biomolecules-10-00090-s001.pdf]

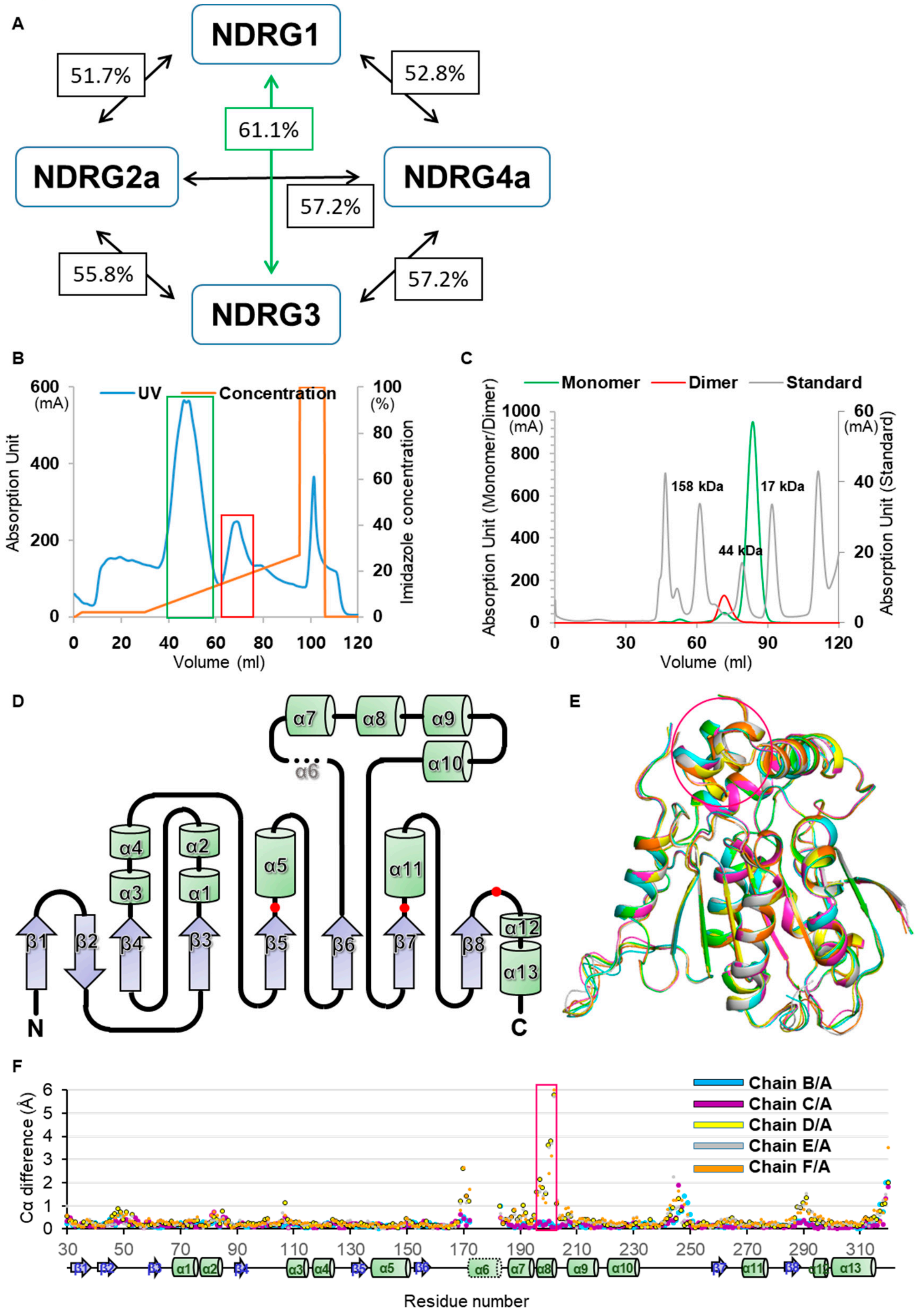

Figure S1. (**A**) Sequence identities among NDRG isoforms. Green arrow represents the highest sequence identity. (**B**) Chromatograms of Nickel-charged immobilized metal chelate affinity chromatography (IMAC) of NDRG3 ΔNC lysate. A green box indicates collected monomer fractions and a red box represents collected dimer fractions, respectively. The orange chromatogram represents the concentration of imidazole. (**C**) Chromatograms of size exclusion chromatography. Chromatograms of monomer and dimer of NDRG3 ΔNC are shown as green and red lines,

respectively. The grey chromatogram indicates a gel filtration standard profile (Bio-Rad #1511901, Hercules, CA, USA). (**D**) Topology diagram of NDRG3. Helices and strands are represented as green cylinder and blue arrow, respectively. The helix α6 region is marked as black dotted line. Red circles indicate the equipositional catalytic triad residues of NDRG3. (**E**) Superimposition of C_α chain of NDRG3 ΔNC subunits in ASU. A red circle indicates helix α8 of NDRG3. The green, cyan, purple, yellow, grey, and orange cartoon models represent chain A, B, C, D, E, and F of NDRG3 ΔNC subunits, respectively. (**F**) Structural comparison of C_α distances between chain A of NDRG3 ΔNC compared with other chains of NDRG3 ΔNC. The secondary structure of NDRG3 is shown at the bottom of the r.m.s.d. distances of Cα atoms.

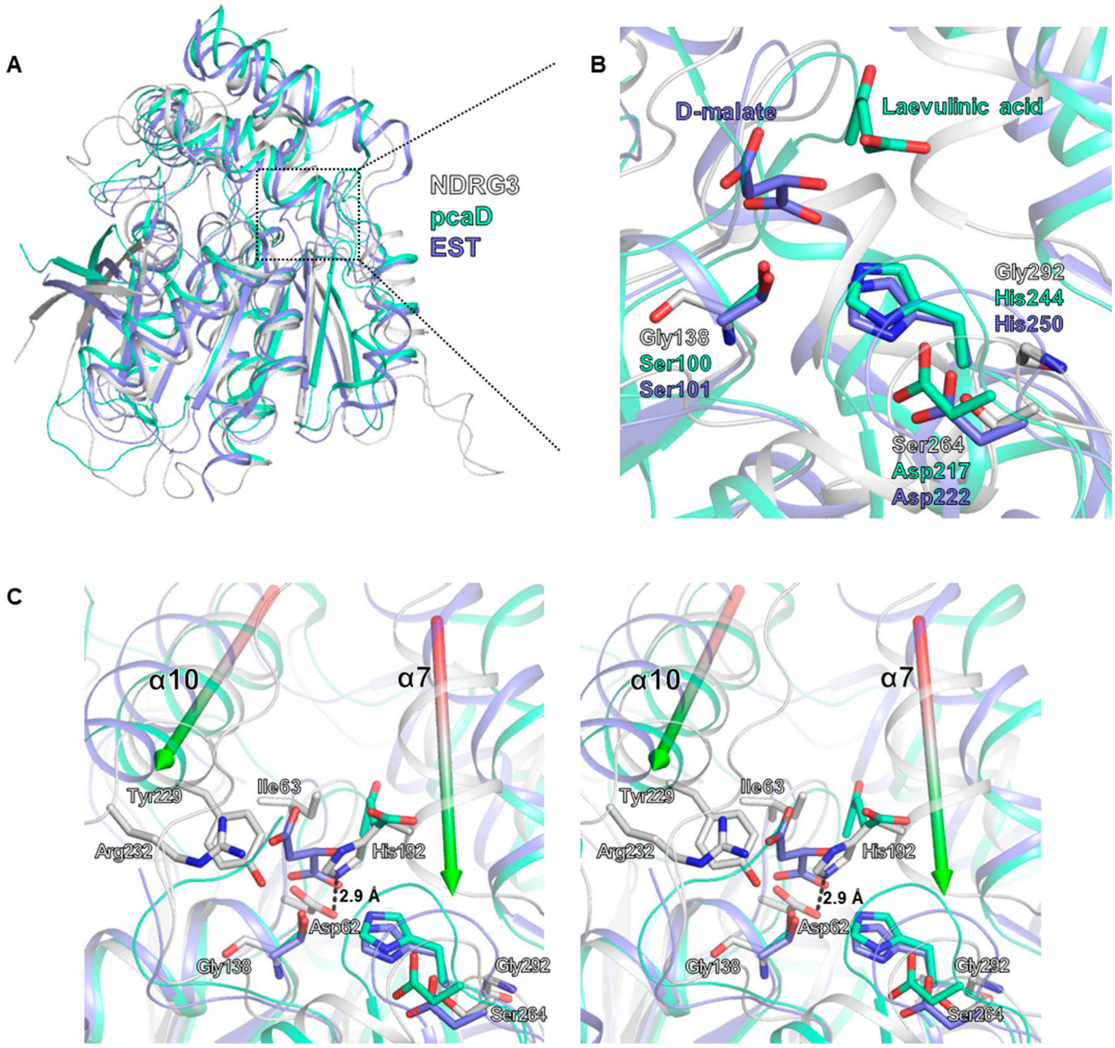

Figure S2. Structural comparison between NDRG3 and its structural homologs, pcaD and EST. (A) Superimposition of Cα chain of NDRG3, pcaD, and EST. Structures of NDRG3, pcaD, and EST are represented as white, cyan, and blue cartoon models, respectively. (B) Close-up view of catalytic active site of α/β-hydrolase proteins. NDRG3, pcaD, and EST proteins are shown in white, cyan, and blue, respectively. Catalytic triads and substrates are represented as stick models. (C) Stereo-view image of active site on which the residues are mapped and denoted as in (B). Arrows indicate the direction of each helix.

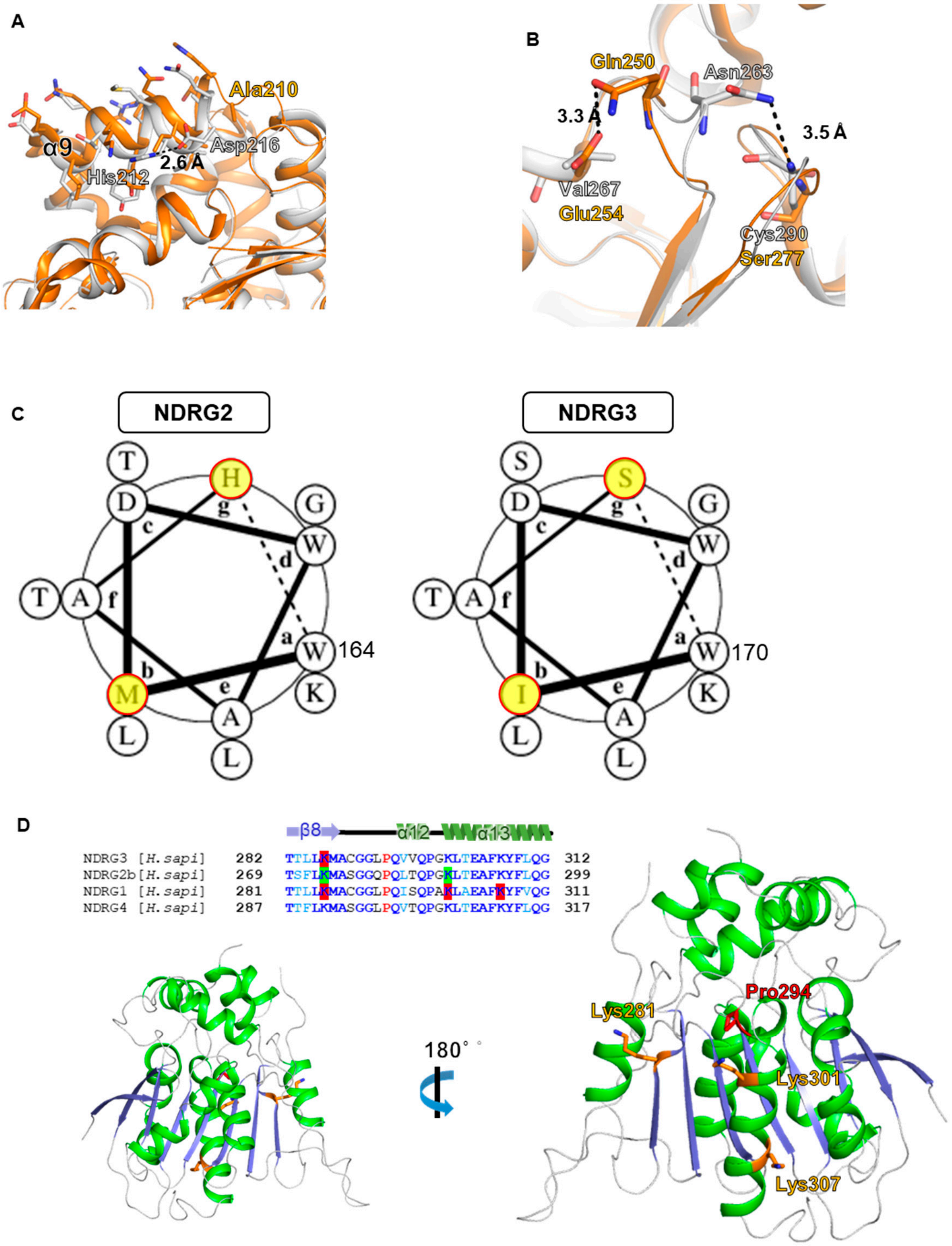

Figure S3. Structural comparison between NDRG3 and NDRG2. (A) Close view of helix α9 region which are marked as red box A in Fig. 3D. White and orange cartoon model represent NDRG3 ΔNC and NDRG2, respectively. Side chains of residue on helix α9 region are shown as stick model. (B) Close view of red circle in Fig. 3D. on which residues are mapped and colored as in (A). (C) Helix wheel model of helix α6 region based on its sequence. Mutated sites are highlighted in yellow. (D) Ubiquitination sites on NDRG3 ΔNC which are analyzed or predicted using the data from the *PhosphoSitePlus* web-server (https://www.phosphosite.org). Sequences near Pro294 of NDRG3 is shown above the crystal structure of NDRG3. Red-shaded box indicates ubiquitination sites analyzed in human cell and green-shaded box represents predicted ubiquitination sites, respectively. Blue and

cyan-colored residues represent the identical and conserved residues among the NDRG isoforms, respectively. Pro294 and ubiquitination sites among NDRG family proteins were represented as red and orange stick models, respectively.

© 2019 by the authors. Submitted for possible open access publication under the terms and conditions of the Creative Commons Attribution (CC BY) license (http://creativecommons.org/licenses/by/4.0/).
